# Cooperation and Shared Beliefs about Trust in the Assurance Game

**DOI:** 10.1371/journal.pone.0144191

**Published:** 2015-12-07

**Authors:** Fredrik Jansson, Kimmo Eriksson

**Affiliations:** 1 Institute for Analytical Sociology, Linköping University, SE-601 74 Norrköping, Sweden; 2 Centre for the Study of Cultural Evolution, Stockholm University, SE-106 91 Stockholm, Sweden; 3 School of Education, Culture and Communication, Mälardalen University, SE-721 23 Västerås, Sweden; Chinese Academy of Science, CHINA

## Abstract

Determinants of cooperation include ingroup vs. outgroup membership, and individual traits, such as prosociality and trust. We investigated whether these factors can be overridden by beliefs about people’s trust. We manipulated the information players received about each other’s level of general trust, “high” or “low”. These levels were either measured (Experiment 1) or just arbitrarily assigned labels (Experiment 2). Players’ choices whether to cooperate or defect in a stag hunt (or an assurance game)—where it is mutually beneficial to cooperate, but costly if the partner should fail to do so—were strongly predicted by what they were told about the other player’s trust label, as well as by what they were told that the other player was told about their own label. Our findings demonstrate the importance for cooperation in a risky coordination game of both first- and second-order beliefs about how much people trust each other. This supports the idea that institutions can influence cooperation simply by influencing beliefs.

## Introduction

A fundamental observation about humans is that we can cooperate to achieve a desired outcome that individuals would not be able to achieve on their own. The scope of this capacity to cooperate appears to be quite unlimited: cooperation may occur in the family and between strangers, in matters big and small, and on scales ranging from dyads to societies [1]. Importantly, though, cooperation does not occur in every possible situation where it would be beneficial. To mention just one example, cooperation failure is often quite spectacular in laboratory experiments where participants need to cooperate to avoid depletion of a common resource [2].

Because of its inherent combination of individual and group-level aspects, the study of why and when cooperation occurs cuts across disciplinary boundaries in social science, although with different emphases. Psychologically and sociologically oriented researchers may focus on how cooperation depends on individual traits like social value orientation or generalised trust (e.g., [3, 4]), or on social identities and group membership (e.g., [5–7]). Political scientists and economists may instead focus on how cooperation is shaped by (boundedly) rational responses to institutions (e.g., [8–10]). These different perspectives complement each other, and ideas from one discipline may inform research in another. The aim of this paper is to develop an idea from the institutional school and subject it to experimental tests.

The political philosopher Brian Skyrms expresses the idea as follows: “the viability of cooperation depends on mutual beliefs, and rests on trust” ([11], p. 2). He traces this idea back to David Hume. Sociologist Diego Gambetta puts it another way: “it is necessary not only to trust others before acting cooperatively, but also to believe that one is trusted *by* others” ([12], p. 216). For political scientist Bo Rothstein, the creation and maintenance of common beliefs (or “common knowledge”) constitute a key pathway through which institutions shape trust and cooperation in society; he expresses the same idea entirely in terms of beliefs: “I believe that you can be trusted if I also believe that you believe that I can be trusted” ([13], p. 84).

The notion that “I believe that you believe that…” is often called a *second-order* belief. Here we will only be concerned with the special case of second-order beliefs where the underlying first-order belief is that someone can be trusted. For this special case we shall use the term *second-order trust*. Rothstein’s idea can then be reformulated as saying that cooperation may rely on second-order trust. The aim of this paper is to subject this important hypothesis to experimental testing.

There seem to be no previous experimental literature examining the impact of higher-order trust on cooperation. However, there are certainly studies on related hypotheses. For instance, Thomas Schelling [14, 15] famously considered pure coordination problems in which two players just wish to choose the same option as the other, regardless of which option this is. He suggested that beliefs about what the other person will do, and beliefs about what the other person believes I will do, and so on, will be important for successful coordination. A recent study in this vein had pairs of participants face the pure coordination problem of choosing the same colour among the options pink, blue and yellow [16]. When information about the other player’s sex was provided, participants tended to use that information and its relation to their own sex to successfully coordinate on a sex-typical or sex-neutral colour.

There are also studies on tacit coordination and cooperation in a volunteers’ dilemma, where the contribution of exactly one player is needed to produce a public good, and in which there is an asymmetrical distribution of the cost of contribution or benefits of the public good. Contrary to predictions from evolutionary [17] and Nash equilibria, actors tacitly coordinate on the strongest group member to punish defectors, thus promoting cooperation [18, 19]. These findings support the importance of second-order social beliefs for successful coordination, but do not speak directly to second-order trust.

Closer to our hypothesis, experimental economists have measured first- and second-order beliefs about others’ contributions in a public goods game [20]. They found that own contributions correlated with both kinds of beliefs, and were affected by the framing of the game. These results are consistent with second-order beliefs being important for cooperation. However, beliefs were not independently manipulated in this study, and therefore only correlational results were obtained. Although the theme was cooperation, a key difference to our hypothesis is that the underlying belief was about contributions, not trust.

Other studies in this vein show that second-order beliefs are predictive of trustworthy behaviour in a game where there is an incentive for the trusted person to defect. Communication and promises of trustworthy behaviour increase mutual cooperation [21]. Rather than being directly caused by beliefs about beliefs, [22] however suggests that the cooperative behaviour in this game may be an effect of a preference for keeping promises.

Another related study dealt with second-order ingroup bias [23]. Participants chose between an unknown monetary allocation made by an ingroup and an outgroup allocator. A strong preference for the ingroup allocator was found only when participants believed the allocator to be aware of their group membership. This finding suggests that preferences for ingroup vs. outgroup members were not caused by direct attribution of positive qualities to ingroup members but rather depended on *expectations* of ingroup members to favour ingroup members, that is, a second-order effect: “I prefer ingroup members because I believe they will give preferential treatment to ingroup members (such as me)”. In other words, this study experimentally demonstrated that ingroup biased behaviour may derive from second-order effects. A more recent study showed that common knowledge of group membership is not only a moderator of ingroup bias, but when both group membership and knowledge about it is shared, both trusting behaviour and beliefs about contributions increase [24]. These findings make plausible, but do not give direct evidence for, the effect of higher-order trust that we examine in this paper.

### A Model of Trust-Based Cooperation

To study the role of higher-order trust—recursive trust—in cooperation we shall use a model situation known as the “assurance game” or “stag hunt”. The latter term comes from a story of Rousseau where two individuals must choose between hunting stag and hare. Hunting stag pays off well if successful, but it will fail if an individual attempts it alone. Hunting hare can be done successfully on one’s own and gives a moderate payoff. The stag hunt is a model that encompasses a whole range of situations where all members of a group will do well if they coordinate on a cooperative endeavour, but where this endeavour will fail if some member defects [7, 11, 25, 26]. Table 1 shows the payoff rules for all combinations of the two players’ choices between the risky strategy to cooperate and the risk-free strategy to defect.

**Table 1:** Payoff matrix of the stag hunt. Note: The table shows payoffs to Ego; to obtain Alter’s payoffs, just switch the roles of Ego and Alter. The numbers within brackets are the payoffs (in SEK) used in the experiments.

	*Alter cooperates*	*Alter defects*
*Ego cooperates*	high [150]	low [0]
*Ego defects*	medium [100]	medium [100]

A game-theoretic analysis shows the stag hunt has two stable Nash equilbria: the *efficient* equilibrium where both players cooperate (receiving a high payoff) and the *risk-dominant* equilibrium where both players defect (receiving a medium payoff). A related game often used in studies of cooperation is the “prisoners’ dilemma”, in which only mutual defection constitutes an equilibrium. Social preferences may transform a prisoners’ dilemma into a stag hunt [27], as can repeated interactions, according to the folk theorem. Other reasons (see also [11]) for why the stag hunt is an important game is that people tend to conceive of prisoners’ dilemmas as stag hunts [28], and it has been shown in theoretical work that the stag hunt may have been more important for ingroup preferences to evolve [29]. One real-world example of a stag hunt with many applications is the problem of deciding whether to devote energy to instituting a new (mutually beneficial) social contract or not. A similar example is whether to reform the social contract, that is, whether to remain in risk-free status quo, or to take the risk of trying to move to a more rewarding equilibrium by improving the social contract ([11], p. 9). Everyday examples include whether to try to arrange to meet with a friend when it might be hard to make an arrangement that both will follow through, or to decide on when to arrive at a party when you want to come as early as possible, but not before some other person(s), or to take part in a desirable action where you might get punished unless everyone is involved.

In order to reason about higher orders of trust in this model of cooperation, let us refer to a decision-making player as “Ego” and the other player as “Alter”. If Ego is rational, then the decision whether to cooperate should depend on whether Ego has sufficient trust in Alter to cooperate. (This statement holds also if Ego holds social preferences that weigh in Alter’s outcome in the decision; this would only change the breakpoint for what level of trust is “sufficient”.) But, for the same reason, Ego should trust Alter to cooperate only to the extent that Ego believes Alter trusts Ego to cooperate. Thus, in this model a rational choice argument supports the hypothesis about the importance of second-order trust that we earlier attributed to Bo Rothstein among others.

Self-referential reasoning is known as recursion. Note that the recursive argument we just gave does not stop at the second order. It is also rational for Ego to cooperate to the extent that Ego believes that Alter believes that Ego trusts Alter to cooperate. Although the recursive argument applies indefinitely in theory, in practice its scope will be limited by individuals’ cognitive capacities. It is not self-evident whether even second-order trust will play a role in actual decisions to cooperate. However, it is not implausible given the related studies of second-order effects reviewed above.

It is the symmetry between the players that makes recursive trust rational. The players face the same payoff matrix (even though it would suffice to have the same ranking of strategies) and the same set of strategies is optimal for both of them. Other important situations lack this symmetry and are fundamentally asymmetric. For instance, the literature on “generalised trust” focuses on asymmetric situations where the players face different strategic choices and optimal outcomes, where someone, the “truster”, can leave herself open to exploitation by someone else, the “trustee” (e.g., [30]). In our symmetric model there is no opportunity for exploitation because players’ interests are aligned.

### Prior Experiments on Cooperation in the Stag Hunt

Although the stag hunt has often been conceived of as a trust problem, we have only found one study that actually measures how trust relates to behaviour in a stag hunt game [3]. Cooperation was not strongly predicted by a measure of generalised trust (a categorisation of participants according to their social value orientation showed that trust had no predictive power at all among selfish individuals but only among prosocial individuals), which is consistent with our symmetry-based argument above. The symmetry argument is also supported by a recent meta-analysis of behaviour in various social dilemmas, which showed that generalised trust becomes less important the smaller the degree of conflict in the social dilemma [31].

Cooperation in the stag hunt is more likely if previous two-way communication is possible [32, 33]. It may be that communication increases a common group identity, which is known to imply both higher trust and more cooperative behaviour [6, 34]. Indeed, [5] found that if participants were divided into minimal groups (e.g., based on “heads” or “tails”) then they cooperated more often in a stag hunt when they knew they were paired with a member of the same group.

### Hypotheses and Outline of Experiments

In two experiments we test predictions from our argument about recursive trust. Specifically, we label participants as “high trusters” and “low trusters” and investigate how information about these labels affects behaviour in the stag hunt. The first prediction says that recursive trust of the second order (i.e., whether Ego believes Alter trusts Ego) is important in decisions on how to behave in stag hunt situations.

***Hypothesis on second-order trust***. Ego should be more likely to cooperate when told that Alter is a high truster (vs. a low truster).

It is crucial to note that this prediction is very different from the standard prediction in the minimal group paradigm. Based on social identity theory, the standard prediction is that participants should show a bias in favour of others with the same label. Thus, high trusters should preferentially cooperate with other high trusters, and low trusters with other low trusters. In contrast, the second-order trust hypothesis says that both high and low trusters will preferentially cooperate with high trusters.

The next prediction says that cooperation will be affected also by third-order trust (i.e., whether Ego believes Alter believes Ego to trust Alter):

***Hypothesis on third-order trust***. Ego should be more likely to cooperate when told that Alter is told that Ego is a high truster (vs. a low truster), regardless of whether Ego is actually high or low on generalised trust.

Note that someone who might actually be high on generalised trust but is told that the other knows him or her as a “low truster” is predicted *not* to cooperate. Thus, the third-order trust hypothesis says that information about information about trust can override both individual differences in actual trust and ingroup effects based on the same information.

We shall test our hypotheses in two experiments. The first experiment assigned participants labels as either high or low trusters based on their responses to some trust items. In the second experiment, participants were instead assigned the labels “high truster” and “low truster” arbitrarily, playing under one label for half the experiment and then switching to the other label. Neither of the experiments involved deception, so in the second experiment participants were truthfully told that labels were assigned randomly. In both experiments participants played a sequence of one-shot stag hunt games with different partners and varying amounts of information on who is a high or low truster. The within-subject design eliminates the effect of individual preferences as subjects are not likely to change their preferences between conditions.

## Experiment 1

### Participants

Two hundred forty-five participants were recruited from a pool of volunteering students at Stockholm University. Eleven were excluded after post-experiment debriefing revealed that they had misinterpreted parts of the game, leaving a total of 234 participants (101 women and 133 men) for the analysis.

### Procedures

Participants were shown to separate cubicles where, using a computer provided, they answered a questionnaire and played a sequence of one-shot stag hunt games with different anonymous partners. Among 19 filler items the questionnaire included four items on generalised trust forming a trust scale previously used by [35]. For each statement, the participant selected a level of agreement from −2 to 2. Two of the questions counted towards (marked with a +) and two against the participant’s trust score (marked with a −). In total, the answers gave a cumulated trust score from −8 to 8. The statements were the following:

- Most people are basically honest (+)
- One should not trust others until one knows them well (−)
- Most people will respond in kind when others trust them (+)
- Those devoted to unselfish causes are often exploited by others (−)

These items are thus not specific to the stag hunt situation, which would be another possible trust measure, but estimate a general inclination to trust others. Generalised trust is easier to distinguish from recursive trust and thus provides a more conservative measure for second- and third-order (but not first-order) trust. Based on a median split of trust scores in each session, participants were labelled either “high trusters” or “low trusters”. Forty-nine participants who scored exactly on the median were arbitrarily labelled. Note that the purpose here is not to provide accurate trust scores and generally appropriate labels of the participants, but to present a label that the subjects would acknowledge as a trust label (that is not void of content or arbitrarily assigned). All participants then played the stag hunt game, with payoffs as in Table 1, in a series of conditions described below. The terms “cooperation” and “defection” were never mentioned in the instructions; the strategies were instead referred to as A and B.

There was never any communication between players, and until the end of the session participants were not given any feedback about any other player’s decisions or any outcome of the games. At the end of the session participants were paid (according to the outcome of one randomly drawn stag hunt game), debriefed and dismissed. There was no deception in the information given.

Ethics approval was obtained from the Regional Ethical Review Board in Uppsala, Sweden. Written informed consent was received from all participants.

### Conditions

To maximise variation in information conditions, different sessions used one of two different sets of conditions. These sets are described in Table 2, referred to as experiments 1A and 1B. The number of stag hunt games played in each information condition depended on whether it included information about the other player’s label. In conditions where such information was given, the game was played once with a low truster and once with a high truster (in random order). In conditions where no information about the other player’s label was given, the game was played just once.

**Table 2:** Conditions and frequencies of cooperation in Experiment 1.

Condition [info to Ego: info to Alter]	Alter’s label	Ego “low”	Ego “high”
Experiment 1A (54 “low”, 59 “high”)			
Both know the other’s label (not their own) [b:b]	Low	35%	29%
	High	54%	68%
Both know both labels [ab:ab]	Low	22%	34%
	High	37%	61%
Experiment 1B (59 “low”, 62 “high”)			
No knowledge of labels	unknown	39%	50%
Both know Ego’s label, only Alter knows Alter’s [ad:abc]	unknown	34%	61%
Both know Alter’s label, only Ego knows Ego’s [abc:ad]	Low	31%	34%
	High	63%	65%

In translation, information on trust labels was presented by the sentence “some of the questions you answered earlier measure how much trust you have in people” followed by one or more of the following statements:

1. Based on your answers you have [low/high] trust in people.
2. Based on your partner’s answers, he/she has [low/high] trust in people.
3. Your partner does not know your trust level. Your partner knows that you have been informed about his/her trust level.
4. Your partner is informed about your trust level. You will not be informed about your partner’s trust level, and he/she knows this.

Note that these conditions are different from the ones used in studies on shared group membership [23, 24]. The common knowledge condition is similar to **ab:ab**, but in their control condition the partner is presented as having no information. Different conditions presented players with different combinations of these pieces of information to players; see Table 2. Participants were paired with low and high trusters in random order. The conditions **ad:abc** and **abc:ad** with asymmetric information (one player’s label is common knowledge) came in random order in experiment 1B, otherwise conditions followed the order in Table 2.

### Results

#### Second-order trust

In all three conditions (**b:b**, **ab:ab** and **abc:ad**) where Ego knew Alter’s label, cooperation was more frequent when Alter was a high vs. low truster, *p* <.001 in each condition (*χ* ^2^ = 25.0, 16.5 and 31.1, respectively), McNemar tests. Averaged across all these conditions, cooperation was chosen 58% of the time with high trusters compared to 31% with low trusters, a difference of 28 percentage points. This suggests a substantial effect of second-order trust.

#### Third-order trust

In conditions where Ego’s trust label was common knowledge, cooperation was more frequent among high trusters, both in the full information condition **ab:ab** with both labels being common knowledge (analysed as number of times Ego cooperated towards the low and high truster, i.e., 0, 1 or 2, dependent on trust level), *p* ≈.017, *U* = 1208, and in the asymmetric condition **ad:abc**, *p* ≈.0027, *U* = 1328, Mann-Whitney U tests. Averaged across these conditions, cooperation was chosen 52% of the time by high trusters but only 31% of the time by low trusters, a difference of 21 percentage points. This suggests a substantial effect of third-order recursive trust.

#### First-order trust

Next we wanted to exclude the possibility that this was just a first-order effect. We compared between subsequent conditions where information on Ego’s trust label changed to common knowledge. The number of times participants chose the strategy associated with their own trust level increased, both from knowing only the other’s label **b:b** to full information **ab:ab** (measured as the increase in label-consistent choices in the latter two conditions versus the former two, giving a number from −2 to 2), *p* ≈.037, *V* = 348, and from **no information** to asymmetric **ad:abc** (giving a number from −1 to 1), *p* ≈.061, *V* = 275.5, (two-sided) Wilcoxon signed rank tests. These results, though statistically not very strong, indicate that third-order trust had an effect beyond that of first-order trust.

**Fig 1:**
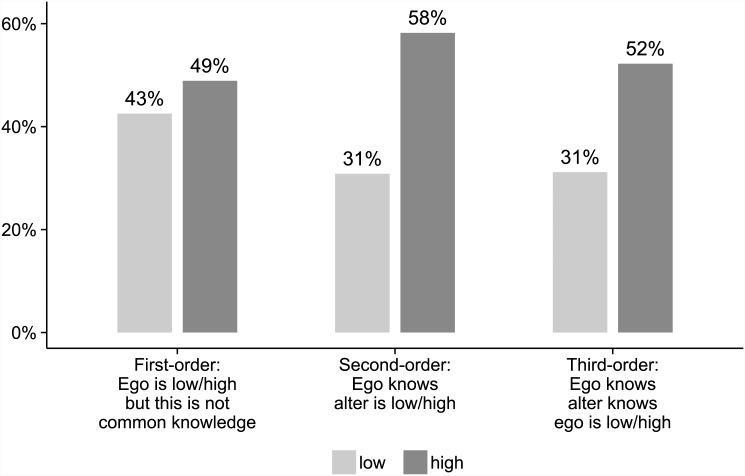
Estimates of effects on cooperation of three orders of recursive trust in Experiment 1.

Averaging across all conditions where the trust level of Ego was *not* known to Alter (**no information** and **abc:ad**) or where Ego does not know what information Alter has (**b:b**), cooperation was chosen 49% of the time by high trusters compared to 43% of the time by low trusters. This is a difference of just 6 percentage points, suggesting that first-order trust was not an important factor in decisions. Fig 1 gives a graphic illustration of the effects of the different orders of recursive trust.

## Experiment 2

A limitation of the first experiment was that third-order trust was only assessed with respect to information about Ego’s “true” trust label. The effect of third-order trust could therefore be distinguished from first-order trust only indirectly. In a second experiment we instead assigned trust labels arbitrarily, and each participant played under both labels.

### Participants

One hundred four participants (48 women and 56 men) were recruited from the same pool of volunteering students at Stockholm University.

### Procedures

Participants were shown to separate cubicles where, using a computer provided, they played a sequence of one-shot stag hunt games with different anonymous partners. Compared to Experiment 1, the game and the procedures were the same. Only the way trust labels were assigned differed between the experiments, as detailed below.

### Conditions

Conditions were the same as in Experiment 1B. After the no-information condition participants were told, in translation, that “all participants have arbitrarily been assigned a ‘trust label’ that describes low or high trust in other people”. Conditions **ad:abc** and **abc:ad** were played with these labels. Participants were then assigned the other trust label and played the same conditions again. The order of trust labels was randomised. Conditions were defined by the following information, and are summarised in Table 3:

ad You are described as having [low/high] trust in other people. Your partner is informed about your trust label. You will not be informed about your partner’s trust label, and he/she knows this.
abc You are described as having [low/high] trust in other people. Your partner does not know your trust label. Your partner’s trust label is that he/she has [low/high] trust in people. Your partner knows that you have been informed about his/her trust level.

**Table 3:** Conditions and frequencies of cooperation in Experiment 2.

Ego’s label	Condition [info to Ego: info to Alter]	Alter’s label	Freq.
none	No knowledge of labels	none	56%
low	Both know Ego’s label, only Alter knows Alter’s [ad:abc]	unknown	25%
	Both know Alter’s label, only Ego knows Ego’s [abc:ad]	Low	25%
		High	61%
high	Both know Ego’s label, only Alter knows Alter’s [ad:abc]	unknown	59%
	Both know Alter’s label, only Ego knows Ego’s [abc:ad]	Low	29%
		High	73%

### Results

#### Second-order trust

In the condition where Alter’s trust label was common knowledge (**abc:ad**), cooperation was chosen more often when Alter was a high truster, regardless of whether Ego was labelled a low or a high truster, both *p* <.001 (*χ* ^2^ = 25.4 and 34.9, respectively), McNemar tests. Averaged across Ego’s both trust labels, cooperation was chosen 67% of the time with high trusters compared to 27% with low trusters, a difference of 40 percentage points. The results were similar also when doing a between-subject comparison for the first round (subjects cooperate towards high trusters 71% of the time, and towards low trusters 26% of the time), showing that ordering is not important.

#### Third-order trust

In the condition where Ego’s trust label was common knowledge (**ad:abc**), cooperation was chosen more often when Ego was assigned the label of high truster, 59%, vs. low truster, 25%, *p* <.001 (*χ* ^2^ = 21.8), McNemar test. This is a difference of 31 percentage points. Again, the results persist also when doing a between-subject comparison for the first round (high trusters cooperate 57% of the time, low trusters 29%), confirming that ordering is not important.

**Fig 2:**
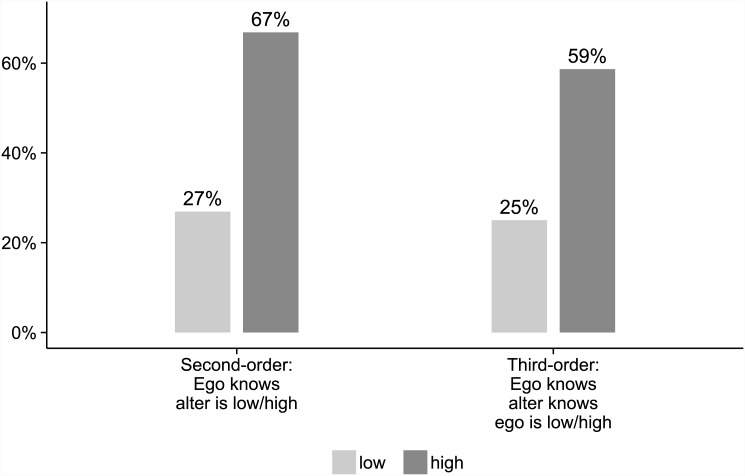
Estimates of effects on cooperation of second- and third-order trust in Experiment 2.

Fig 2 gives a graphic illustration of the estimated effects of second- and third-order trust in this experiment.

## Discussion

Our experiments gave very strong support for recursive trust influencing cooperation in the assurance game. The effect of second-order trust was a 30–40 percentage point difference in the frequency of cooperation depending on whether the first player knew the other player to be a “high truster” or a “low truster”. The effect of third-order trust was a similar 20–30 percentage point difference depending on whether the first player knew the other player knew the first player to be a “high truster” or a “low truster”. Importantly, similar results were obtained whether trust labels were based on a generalised trust scale (Experiment 1) or arbitrary (Experiment 2). The latter finding was important to confirm that the third-order trust effect cannot be explained by standard first-order trust. This was indicated already in the first experiment where the effect of first-order (generalised) trust was weak, consistent with prior findings [3], but the second experiment showed third-order trust also where first-order effects were absent by design.

Our experiment on arbitrary assignment of trust labels could be regarded as belonging to the minimal group paradigm, a very popular and successful manipulation in experimental social psychology. The stag hunt is also a game where an ingroup bias in minimal groups is particularly liable to occur in evolutionary dynamics [29]. Recall the experiment of [5], in which a minimal group manipulation triggered participants to more frequent cooperation with players with the same label. The interpretation from social identity theory is that even the random assignment of labels creates ingroup and outgroup relationships; cooperation is more benevolent towards the other player and is therefore used more when playing with ingroup members. From our experiment we can conclude that the labels “high truster” and “low truster” induced a recursive trust effect that overrode the ingroup effect.

Given that participants cooperated more if and only if the common information was high trust, when labels were assigned arbitrarily, these labels did function as social cues for tacit coordination. In our experiment, however, one of the strategies is both more risky and socially desirable than the other, and rests not only on arbitrary cues, but specifically on signals of trust. Contrasting to a pure coordination game, the definition of the game already includes a cue in that one of the strategies is optimal for both players. Still, without further information, only about half of the participants trusted the other to choose that strategy. While a minimal group manipulation can strengthen the cue within groups, we imposed qualitative labels that do not rely on common group membership and that can work both ways. We have shown that trust labels are sufficient both to increase and decrease actual recursive trust. However, it remains to be investigated whether it is necessary to formulate labels in terms of trust and whether the results would carry over to games that do not rely on trust. Further research could address the first issue by using labels that are qualitatively different with respect to an aspect that is irrelevant for the game in question, and the second by imposing labels of high and low trust in games where trust levels are irrelevant. Here, trust labels were given. Apart from studying the span of effects and formats of trust labels, we also call for studies on how such reputation is created in the first place.

In experimental work on institutions and cooperation, much attention has been paid to the role of rules and sanctions of transgressions (e.g., [9, 36, 37]). Our findings support the argument that there is also another way in which institutions may promote cooperation, at least as long as they resemble a stag hunt: by creating shared beliefs and common knowledge [10, 13]. It appears that such institutions, on the societal macro-level, indeed have the potential to induce recursive trust to the extent that individual interactions, on the micro-level, result in higher cooperation. In order to complete the pathway (in terms of James Coleman’s famous diagram, [38]) from micro- to macro-level outcomes, we might hypothesise that the increased cooperation will transform into more efficient institutions, which may in turn have even more success in creating shared beliefs about generalised trust, thus resulting in a feedback loop. Future research should investigate how cooperation between individuals affects shared beliefs about trust, establishing possible transformations from the micro- to the macro-level.
